# Enhancing Epitranscriptome Module Detection from m^6^A-Seq Data Using Threshold-Based Measurement Weighting Strategy

**DOI:** 10.1155/2018/2075173

**Published:** 2018-06-14

**Authors:** Kunqi Chen, Zhen Wei, Hui Liu, João Pedro de Magalhães, Rong Rong, Zhiliang Lu, Jia Meng

**Affiliations:** ^1^Department of Biological Sciences, RCPM, URCHT, Xi'an Jiaotong-Liverpool University, Suzhou, Jiangsu, 215123, China; ^2^Institute of Ageing & Chronic Disease, University of Liverpool, L7 8TX, Liverpool, UK; ^3^School of Information and Control Engineering, China University of Mining and Technology, Xuzhou, Jiangsu, 221116, China; ^4^Institute of Integrative Biology, University of Liverpool, L7 8TX, Liverpool, UK

## Abstract

To date, with well over 100 different types of RNA modifications associated with various molecular functions identified on diverse types of RNA molecules, the epitranscriptome has emerged to be an important layer for gene expression regulation. It is of crucial importance and increasing interest to understand how the epitranscriptome is regulated to facilitate different biological functions from a global perspective, which may be carried forward by finding biologically meaningful epitranscriptome modules that respond to upstream epitranscriptome regulators and lead to downstream biological functions; however, due to the intrinsic properties of RNA molecules, RNA modifications, and relevant sequencing technique, the epitranscriptome profiled from high-throughput sequencing approaches often suffers from various artifacts, jeopardizing the effectiveness of epitranscriptome modules identification when using conventional approaches. To solve this problem, we developed a convenient measurement weighting strategy, which can largely tolerate the artifacts of high-throughput sequencing data. We demonstrated on real data that the proposed measurement weighting strategy indeed brings improved performance in epitranscriptome module discovery in terms of both module accuracy and biological significance. Although the new approach is integrated with Euclidean distance measurement in a hierarchical clustering scenario, it has great potential to be extended to other distance measurements and algorithms as well for addressing various tasks in epitranscriptome analysis. Additionally, we show for the first time with rigorous statistical analysis that the epitranscriptome modules are biologically meaningful with different GO functions enriched, which established the functional basis of epitranscriptome modules, fulfilled a key prerequisite for functional characterization, and deciphered the epitranscriptome and its regulation.

## 1. Introduction

In the exploration of epigenetic modifications of RNA that has lasted for 5 decades, more than 100 types of posttranscriptional chemical RNA modifications have been identified [1]. Among these modifications,* N*^6^-methyladenosine (m^6^A) is the most abundant type of RNA modifications that steers or participates in various biological functions including circadian clock [2], translation [3, 4], cortical neurogenesis [5], microRNA processing [6], Drosophila sex determination [7, 8], T cell homeostasis [9], RNA-protein interaction [10], and RNA stability [11, 12]. It also plays an important role in DNA damage response [13], heat shock response [14], and the resolution of naïve pluripotency towards differentiation [15]. As RNA methylation participates in many fundamental cellular processes, it is closely related to many types of disease, such as cancer [16, 17] and virus infection [18]. It has been shown that m^6^A demethylase ALKBH5 maintains the tumorigenicity of glioblastoma stem-like cells by programming cell proliferation [19]; the m^6^A demethylase FTO plays as an oncogene in Acute Myeloid Leukemia [20]; and the m^6^A methyltransferase METTL3 controls myeloid differentiation of normal hematopoietic and leukemia cells [21]. Mutations of the RNA methylation enzymes are linked to colon cancer and endometrial cancer [22]. Due to the importance of RNA m^6^A modification to biological regulation and health, it is of crucial importance and increasing interest to study how the epitranscriptome is shaped to regulate relevant biological processes.

There are a large number of RNA m^6^A sites enriched near stop codon, on 3'UTRs and on long exons of the transcriptome [23]. It was originally reported in 2012 that there exist over 12,000 m^6^A sites on 7676 mammalian genes that contain m^6^A [24, 25]. Due to the limitation of sequencing depth, context-specific expression, and dynamics of RNA m^6^A sites, the actual number of m^6^A sites in the human epitranscriptome is likely to be much larger. There are more than 0.3~0.4 million predicted unique m^6^A sites reported in the human epitranscriptome according to two recent bioinformatics databases RMBase [26] and MetDB [27], which are collected by merging MeRIP-Seq data from published studies, although many of these m^6^A sites may exist under very few conditions (tissue/cell types/treatment) or even false positive due to the way the sites are searched; i.e., an unmodified a residual, which conforms the RRACH motif, was false positively reported by the MeRIP-Seq technique due to its proximity to real m^6^A sites [24, 25].

The m^6^A modification is directly deposited or erased by relevant enzymes, i.e., RNA m^6^A methyltransferase (writer) and demethylase (eraser), which are accountable to the observed landscape of m^6^A epitranscriptome in cells. The most well studied m^6^A methyltransferase is a complex [28–30] composed of at least four proteins, including METTL3, METTL14, WTAP, and KIAA1429 [29, 31–33]. It has been shown that METTL3 functions catalytically, while the other proteins mainly serve as regulatory units that mediate the substrate specificity of the methyltransferase complex [34–36]. The fat mass and obesity associated protein (FTO) was identified in 2011 as the first known m^6^A demethylase [37]. Moreover, the protein ALKBH5, derived from the same protein family (ALKB) of FTO, was identified as a second m^6^A demethylase that impacts RNA metabolism and mouse fertility [38]. Very recently, METTL16 is identified as another RNA m^6^A writer that targets pre-mRNAs and noncoding RNAs [39].

Although there are likely to be additional m^6^A-relevant enzymes yet discovered by people, the total number of primary m^6^A-regulating genes is likely to be much less than the total number of m^6^A sites in the epitranscriptome. Due to the substrate specificity of m^6^A-relevant enzymes, epitranscriptome modules are naturally formed when a larger number of m^6^A sites are regulated by a small number of regulators; i.e., the m^6^A sites that share the same regulator will exhibit similar methylation pattern across different experiment conditions, reflecting the catalytic efficacy of their common regulator under respective conditions. The concept of regulatory module has been used extensively in the field of bioinformatics. For example, a transcriptional module of 148 genes that are downregulated during differentiation has been functionally associated with self-renewal [40]. A transcriptional module of 4382 genes is identified to be associated with cell cycle from a time course data with 24 samples in yeast using state space models [41]. In DNA methylation data analysis, modules in the epigenome have been associated with ageing effects [42] and alcohol use disorders [43]. In studies of lncRNA, coexpression of a gene and an lncRNA is often a strong indication for functional relevance of the two and has been used for predicting the functions of novel lncRNAs [44, 45]. Given the aforementioned examples in transcriptomics, epigenomics, and genomics, because the methylation sites of the same epitranscriptome module are coregulated across different experiment conditions, it is reasonable to speculate that they are functionally related as well, i.e., participating in the same or related biological processes and pathways.

Previously, the studies of epitranscriptome module are mainly restricted to the study of substrate specificity of the epitranscriptome enzymes. Through the perturbation of m^6^A writers, Regev lab identified two distinct classes of m^6^A sites based on whether they depend on WTAP, a key regulator of the METTL13-METTL14 writer complex [33]. Liu et al. performed four different clustering approaches to 3274 preselected RNA methylation sites and identified an epitranscriptome module that is likely to be mediated by the m^6^A demethylase FTO [46]. As increasing significance and biological functions of RNA m^6^A modifications are revealed by recent studies, it is of growing necessity to understand the epitranscriptome regulation. The study of epitranscriptome modules provided a viable venue to achieve it.

Currently, the most popular high-throughput sequencing approach for profiling RNA methylome is methylated RNA immunoprecipitation sequencing (MeRIP-seq or m^6^A-seq) [24, 25]. From technical perspective, m^6^A-seq may be considered as a marriage of RNA-seq and ChIP-seq technique, where the methylation signal is obtained by sequencing the immunoprecipitated RNA fragments with anti-m^6^A antibody (the IP sample), and the control background is generated using all the input RNA fragments (the input sample). A major difficulty faced by computational biologists when searching for the epitranscriptome modules is to deal with the artifacts in epitranscriptome high-throughput sequencing data, which is mainly due to the context-specific gene expression, the limitation of sequencing depth. Constrained by the detection ability, it is always very difficult to accurately quantify the methylation level of very lowly expressed genes. For example, if the reads count of a specific methylation site in the IP sample is *t* and the reads count in the paired input sample is *c*, without taking into account the difference in sequencing depth, a natural measurement for the methylation level of this site *m* is

(1) $$\begin{array}{c} m=\frac{t}{t+c}, \end{array}$$

where *m* ∈ [0,1]. This way of quantifying methylation level has been widely used in DNA methylation analysis in the form of beta-value [47]. However, this approach can be problematic in RNA methylation data analysis when dealing with very lowly expressed genes. For example, while a methylation site with *t* = 100 and *c* = 0 is likely to be highly methylated (*m* = 1) a methylation site with *t* = 1 and *c* = 0 may not (*m* = 1). As a matter of fact, there is barely any signal for the latter case to make any reliable estimation, although the estimated methylation level is 1. Different from DNA methylation data, where the background is homogenous and the background reads coverage is expected to be the same across the entire genome, there exists rather prominent heterogeneity in the reads coverage of the transcriptome and epitranscriptome data; i.e., there are usually a small number of highly expressed genes coupled with a very large number of lowly expressed genes, whose methylation signal is too weak to be estimated reliably, which severely limits the performance of computational approaches based on this measurements. It is necessary to develop strategy that can take advantage of the estimated methylation level together with its reliability.

To unlock the full potentials of epitranscriptome sequencing data, we designed a convenient measurement weighting strategy to incorporate the measurements together with their reliability as the weight. Under this scheme, unreliable measurements that are supported by relatively small number of reads are given less weight in the computation model, while measurements supported by a large number of reads are given more weight. In this way, even if some measurements are not accurate, because smaller weights are assigned to them, the final computation results are still likely to be robust. We will show in the next how to use the weighting strategy in a hierarchical clustering approach to find epitranscriptome modules with weighted Euclidean distance and show the performance improvement compared with the same method but without using the weighting scheme.

## 2. Method

Considering we have the methylation profile of *N* methylation sites obtained from *S* experimental conditions and the reads count of the *n*-th methylation site under *s*-th condition in the IP sample is *t*_*n*,*s*_, the reads count of the *n*-th methylation site under *s*-th condition in the input sample is *c*_*n*,*s*_. The size factors of the IP and input samples of the *s*-th condition are *d*_*s*,*t*_ and *d*_*s*,*c*_, respectively, which reflects the sequencing depth (or library size) of the sample, which may be estimated using geometric mean or other approaches. Based on previous definition, the estimated methylation level of the *n*-th methylation site under *s*-th condition is

(2) $$\begin{array}{c} m_{n,s}=\frac{t_{n,s}/d_{s,t}}{t_{n,s}/d_{s,t}+c_{n,s}/d_{s,c}}=\frac{t_{n,s}d_{s,c}}{t_{n,s}d_{s,c}+c_{n,s}d_{s,t}} \end{array}$$

where *n* ∈ {1,2, ⋯, *N*} and *s* ∈ {1,2, ⋯, *S*}. When give the estimated RNA methylation profiles, it is fairly easy to apply hierarchical clustering approach to search for epitranscriptome modules. A typical measurement people use to measure the similarity of two methylation profiles is the Euclidean distance, where the distance between the *i*-th and *j*-th methylation sites *d*(*i*, *j*) may be calculated as follows:

(3) $$\begin{array}{c} d\left(\;i,j\;\right)=\sqrt{\overset{S}{\underset{s=1}{\sum }}\left[{\left(m_{i,s}-m_{j,s}\right)}^{2}\right]} \end{array}$$

Smaller *d*(*i*, *j*) suggests that the two sites share a very similar methylation profile across different experimental conditions, belong to the same epitranscriptome module, may be regulated by the same epitranscriptome regulator, and may be functional relevant based on previous experience in genomics analysis. However, the distance measurement by Euclidean distance can be seriously affected by a few unreliable measurements estimated from a small number of reads and then may seriously jeopardize the clustering results. To fully take advantage of the potentials of the epitranscriptome sequencing data, we consider here a weighting strategy by using the weighted Euclidean distance. Specifically, the weighted Euclidean distance between the *i*-th and *j*-th methylation sites *d*_*w*_(*i*, *j*) may be calculated as follows:

(4) $$\begin{array}{c} d_{w}\left(\;i,j\;\right)=\sqrt{\overset{S}{\underset{s=1}{\sum }}\left[w_{s,i,j}{\left(m_{i,s}-m_{j,s}\right)}^{2}\right]} \end{array}$$

where *w*_*s*,*i*,*j*_ > 0 and ∑_*s*=1_^*S*^*w*_*s*,*i*,*j*_ = 1. The weight is determined by a function of the reads counts *w*_*s*,*i*,*j*_ = *f*_*w*_(*t*_*i*,*s*_, *c*_*i*,*s*_, *t*_*j*,*s*_, *c*_*j*,*s*_). In this formulation, the weight assigned to the *s*-th experimental condition *w*_*s*,*i*,*j*_ should reflect the reliability of this sample. If the measurements obtained under this sample were estimated from a small number of reads (*t*_*i*,*s*_, *c*_*i*,*s*_, *t*_*j*,*s*_, *c*_*j*,*s*_), the relevant part of the result may not be reliable and a smaller weight should be assigned.

Although it is conceptually easy to depict the desired properties of the weight function *w*_*s*,*i*,*j*_ = *f*_*w*_(*t*_*i*,*s*_, *c*_*i*,*s*_, *t*_*j*,*s*_, *c*_*j*,*s*_), there still exist different ways to define the function and it is still an open question how to choose a proper weighting strategy according to the data. In this manuscript, we consider the following 2 weighting schemes:

(i) logarithm-based approach, in which,
  (5) w~s,i,j=fwti,s,ci,s,tj,s,cj,s=log⁡ti,s+ci,s+tj,s+cj,s+1
  (6) ws,i,j=w~s,i,j∑∀sw~s,i,j
In this approach, the weight of a sample increases logarithmically with the number of reads. Conceivably, it is reasonable to assume that there exists a big difference in terms of reliability between measurements supported by 2 and 200 reads but only very minor difference between those supported by 1002 and 1202 reads.
(ii) threshold-based approach, in which
  (7) w~s,i,j=fwti,s,ci,s,tj,s,cj,s=1whenti,s+ci,s+tj,s+cj,s≥αβwhenti,s+ci,s+tj,s+cj,s<α
  (8) ws,i,j=w~s,i,j∑∀sw~s,i,j
This approach is conceptually similar to the “threshold method” [52] with two parameters *α* and *β*. When the total number of reads mapped to the two sites in sample *s* is greater than the threshold *α*, we consider they are fairly accurate measurements and assign a normal weight 1, while a smaller weight *β* is assigned to the sample when the measurements of methylation level are not reliable, with 0 ≤ *β* ≤ 1. In practice, it is necessary to further optimize the two parameters *α* and *β ad hoc *with respective to the datasets used.

With two different measurement weighting strategies defined as previously, we will next compare the proposed approach on real data with conventional approach without measurement weighting.

## 3. Result

### 3.1. RNA Methylation Sequencing Data

The datasets used in the following analysis were from published studies and downloaded directly from Gene Expression Omnibus (GEO) in SRA format. The data profiles the m^6^A epitranscriptome in HEK293, HepG2, U2OS, and human brain under different treatments (see Table 1). The reads are aligned to human reference genome assembly (hg19) with the default setting of Tophat2 [53]. Subsequently, the epitranscriptome (all the RNA m^6^A methylation sites under different conditions) was retrieved using exomePeak [54] with UCSC gene annotation and default settings by following a previous approach [46]. Furtherly, the reads count of every RNA methylation site (*t*_*n*,*s*_ and *c*_*n*,*s*_ for ∀*n* ∈ {1,2, ⋯, *N*} and ∀*s* ∈ {1,2, ⋯, *S*}) is retrieved using R/Bioconductor packages [55]. The biological replicates obtained from the same condition are merged together, and the total number of reads is used to estimate the size factor of samples (*d*_*s*,*t*_ and *d*_*s*,*c*_ for ∀*s* ∈ {1,2, ⋯, *S*}). The methylation ratio of all sites is estimated according to (2). The estimated methylation level is then quantile normalized to remove possible batch effect. Only the sites that show strong dynamics are retained for further analysis; this is achieved by selecting the RNA methylation sites with larger variance in methylation level. Finally, the methylation profile of each methylation site is standardized by subtracting the mean and divided by its standard deviation to ensure all sites contribute equally to the analysis.

### 3.2. Comparing Different Weighting Schemes Using True Sample Labels

A major limitation for assessing the performance of different weighting schemes in a clustering approach for RNA methylation analysis is the lack of ground truth. The epitranscriptome regulation is complicated, and it is not clear which group of RNA methylation sites shares a common regulator across different experimental conditions. To this end, an alternative approach is considered by taking advantage of the sample labels. There are 6 samples including two triplicates profiling the m^6^A epitranscriptome in human U2OS cell line with or without DAA treatment (see Table 1, Dataset ID 8 & 9), and if a clustering approach is applied to the 6 samples, it should retrieve two distinct groups corresponding to the DAA and control conditions in the experiment setting. Conceivably, when two different weight schemes are used in the clustering analysis, the one that returns more consistent results with experimental setting suggests a better performance.

The sample label is used as group truth for clustering analysis in the first experiment. Specifically, a total of 916 small datasets, each containing the methylation profiles of 6 samples and 30 RNA methylation sites adjacent with each other in genomic coordinates, are generated by splitting the original high-throughput dataset (Dataset ID 8 & 9 in Table 1), and a hierarchical clustering classifier using Euclidean distance with different weighting schemes (no weighting, logarithm-based and threshold-based) was applied to the small datasets to group the samples into 2 clusters, and the clustering results are then compared to the true sample labels for assessing the clustering performance on all the 916 small datasets. In this analysis, the parameters of threshold-based approach (*α* and *β*) were arbitrarily set to 0.03 to 0.45, respectively, without necessary optimization. Instead of using a specific threshold value for *β*, we use here a relative quantile value, where 0.45 is corresponding to the 45% quantile for reads count of all the measurements. As is shown in Table 2, given that there are a total of 6 samples from groups, the probability to obtain a correct clustering result by random is only 3.2%. The RNA methylation profile contains clustering information. Correct clustering results may be obtained for more than 26% of times when the standard approach is applied, which assigns all measurements with equal weight. Additionally, the clustering performance can be further improved by taking advantage of the proposed weighting strategy (32% and 37.6%), which shows the proposed measurement weighting scheme can significantly improve the clustering performance. Please note that the correct percentage is relative low because we used a very stringent criteria; i.e., the clustering results are considered correct if and only if all the samples are clustered correctly. These performances are about ten times more accurate than that achieved from a random classifier (3.22%), which suggests that the clustering results are statistically meaningful. Additionally, we show in Supplementary Materials that the proposed threshold-based weighting scheme is equally applicable when using M-value to quantify the RNA methylation status (see Table S1) and it is also useful when using squared Euclidean distance or City Block to measure the similarity of RNA methylation profiles (see Tables S2 and S3).

**Figure 1:**
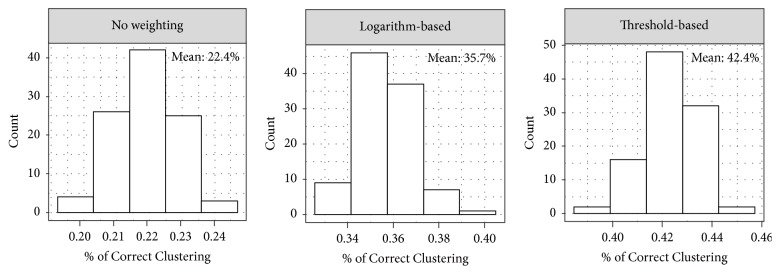
**Performance of method using or not using measurement weighting strategy**. When datasets of 30 RNA methylation sites and 6 samples are using for clustering analysis, the proposed two weighting strategies always lead to performance improvement.

In the previous result, the 916 small datasets were generated by splitting the complete high-throughput dataset, and the RNA methylation sites of the same small dataset are adjacent to each other on the genome, which may possess systematic correlation that influences the clustering result. To eliminate this bias, we consider a more random test in the next. Specifically, 916 small datasets with 30 random selected methylation sites are generated, to which clustering analysis using different weighting strategies was applied and the clustering performance was assessed again using the true sample labels. The analysis was repeated for 100 times, and the results are shown in Figure 1. The proposed weighting strategies consistently improve the clustering performance. We also tested the cases when M-value, squared Euclidean, or City Block is used to quantify the RNA methylation status or the similarity of RNA methylation profiles. As is shown in Supplementary Materials Figure S1, consistent improvement in clustering performance is observed when the proposed weight scheme is implemented.

**Figure 2:**
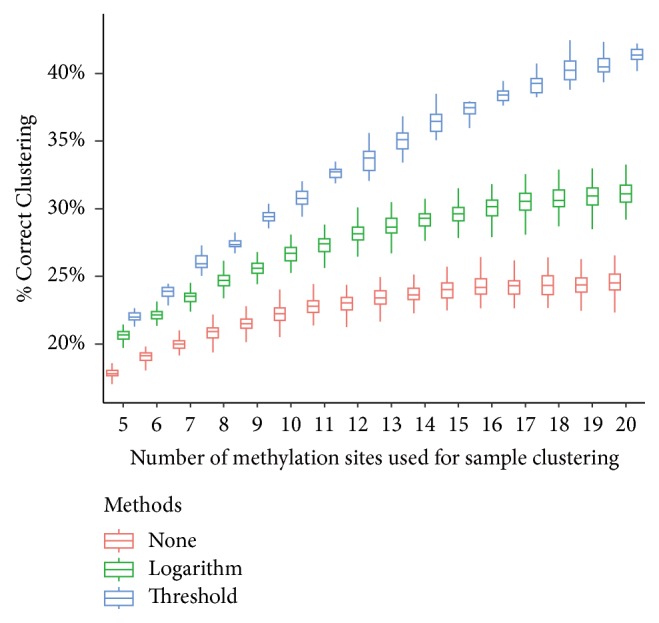
**Impact of dimension size**. Small datasets of different number of RNA methylation sites are generated, to which the clustering approach was applied with or without sample weighting strategies. The clustering performance increases as the dimension (number of RNA methylation sites) increases, and the clustering method using the weighting strategies consistently outperforms the one that does not use it.

In the previous study, we tested a case when there are 30 RNA methylation sites available for the clustering analysis. We study next the influence of dimension size on clustering performance by changing the number of sites included in the analysis. As shown in Figure 2, the clustering performance increases as the dimension (number of RNA methylation sites) increases, and the clustering method using the weighting strategies consistently outperforms the one that does not use it. In all setting tested, threshold-based weight strategy provides the best clustering performance and the logarithm-based weighting strategy also outperforms the one that does not use measurement weighting. It is now rather clear that many measurements are not accurate and need to be penalized in some way in the analysis. We also tested the cases when M-value, squared Euclidean, or City Block is used to quantify the RNA methylation status or the similarity of RNA methylation profiles. As is shown in Supplementary Materials Figure S2, very similar results are observed. The proposed approach can consistently improve clustering performance when different quantification methods or distance measurements are used.

### 3.3. Parameter Optimization for Threshold-Based Weight Strategy

**Figure 3:**
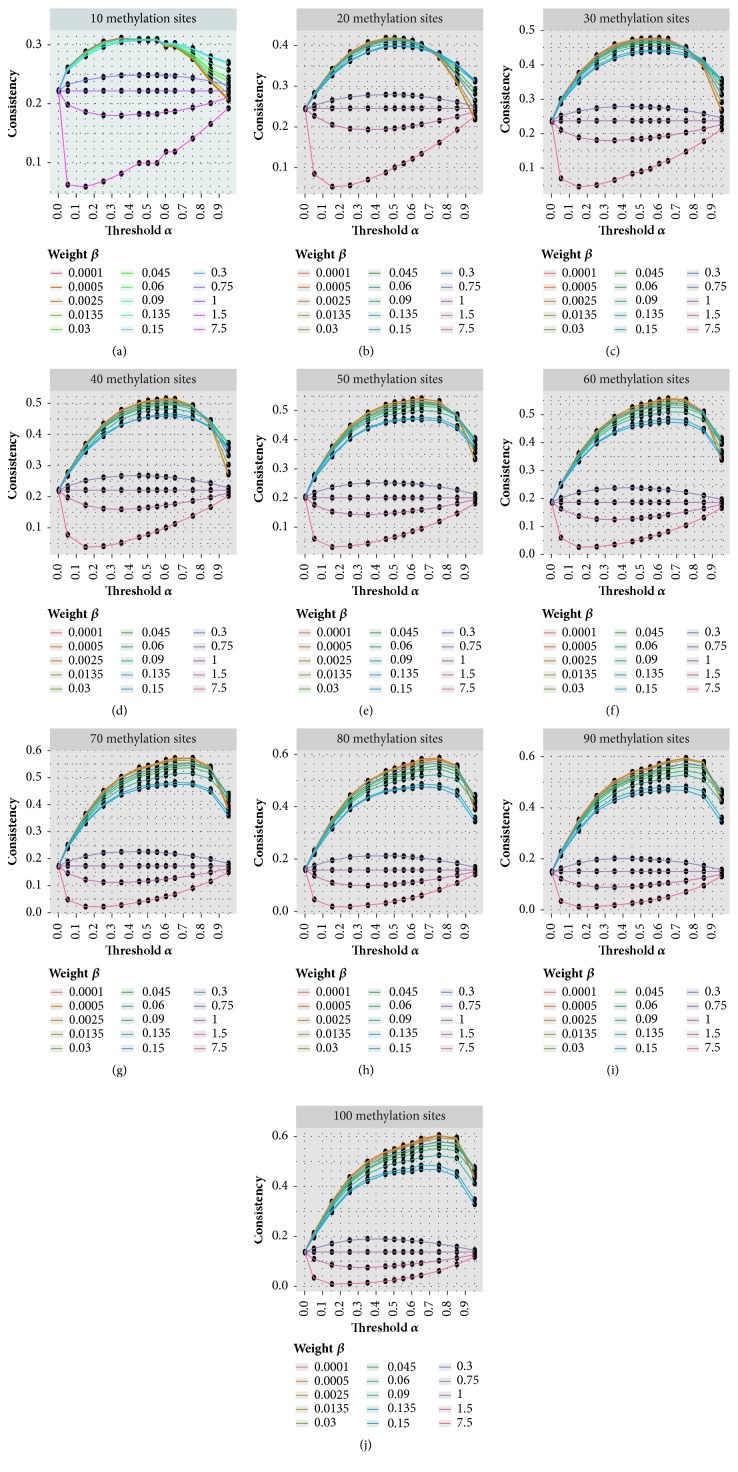
**Parameter optimization for the threshold-based method**. Small datasets are generated by sampling randomly from real RNA methylation dataset, to which clustering analysis used the threshold-based weighting strategy with different parameters and the clustering performance was evaluated by comparing to true sample labels. Better clustering performance was achieved when setting a relative small value for weight parameter *β* and a medium value for threshold parameter *α*.

With previous results, the threshold-based measurement weighting strategy has shown superior performance; this method will be our focus in the next section. It is worth mentioning that the parameters of this method; i.e., the threshold *α* and weight *β* are still not sufficiently optimized. It is important to further fine-tune these two parameters for the best possible performance. Till this end, we consider here a 2-D grid search, where all combinations of *α* and *β* are tested, with the threshold parameter ∈[0, 0.05, 0.15, 0.25, 0.35, 0.45, 0.50, 0.55, 0.65, 0.75, 0.85, 0.95] and the weight parameter *β*∈ [1E-4, 5E-4, 2.5E-3, 1.35E-2, 0.03, 0.045, 0.06, 0.09, 0.135, 0.15, 0.3, 0.75, 1, 1.5, 7.5]. Please note that when *α* = 0 or *β* = 1, no measurements will be penalized and the weighting strategy will be essentially the same as standard approach without measurement weighting. When *β* > 1, a larger weight will be assigned to the measurements that are less accurate, which is expected to damage the clustering performance. This setting is used in the analysis as a negative control. Similar to before, the performance is tested on small random datasets with different number of RNA methylation sites (*N*∈[10, 20, 30, 40, 50, 60, 70, 80, 80, 90, 100]). After repeating the analysis 100 times, the average clustering performance under each possible combination of setting is summarized in Figure 3. We can see that the performance patterns on dataset of different sizes are similar. Better clustering performance was achieved when setting a relative small weight parameter *β* and a medium threshold parameter *α*. A large weight parameter *β*, which assigns a larger weight to less accurate measurement, always undermines the clustering performance, just as previously expected. When comparing the results achieved on datasets of different size, the optimal threshold parameter *α* increases as the data size increases. This observation is reasonable, because compared with small dataset, a larger dataset can afford to lose more unreliable measurements in the analysis.

### 3.4. Quality Assessment of Epitranscriptome Modules with Gene Ontology Analysis

We demonstrated with previous analysis the effectiveness of measurement weighting strategy in clustering analysis of biological samples by referring to the sample labels as the ground truth. It is important to test whether the proposed measurement weighting strategy is equally useful in the search of epitranscriptome modules, i.e., clustering the RNA methylation sites into different groups where the sites belong to the same group show consistent hyper- or hypomethylation states under different experiment conditions, suggesting the sharing of a common regulator.

There are a total of 42,758 methylation sites identified from 9 experimental conditions in the datasets. Data preprocessing was firstly performed, in which we aim to select the assured RNA methylation sites with substantial dynamics in the methylation level across different experimental conditions. We selected the top 20,000 RNA methylation sites with the largest average methylation level and then the top 10,000 sites with the largest variance in methylation level among the previously selected sites. These sites show strong methylation signal and strong dynamics in the data analyzed, which are likely to capture the epitranscriptome modules induced by epitranscriptome regulators.

**Figure 4:**
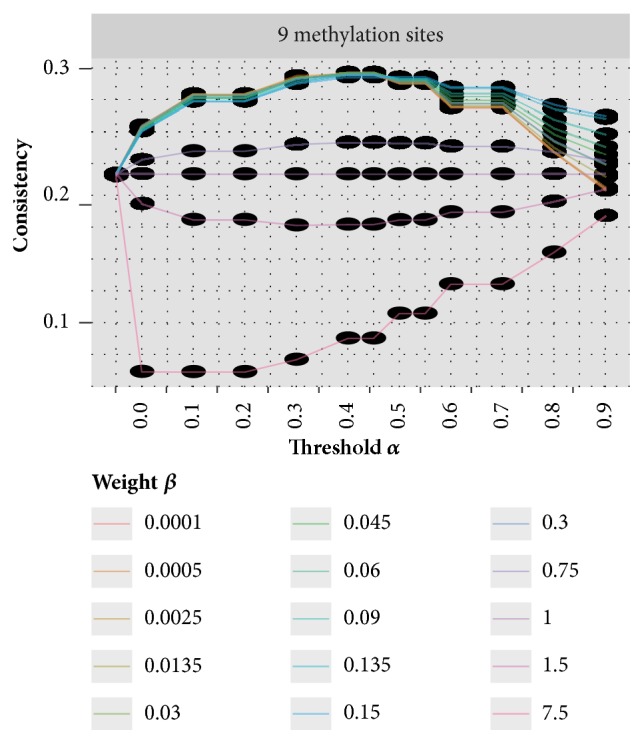
**Parameter optimization for the threshold-based method on real data**. Small datasets of the same dimension size as real dataset were generated, to which clustering analysis used the threshold-based weighting strategy with different parameters. Optimal clustering result on a dataset of 9 measurements is achieved when setting *α* = 0.45 and *β* = 0.09, which will be adopted in the following clustering analysis on real data.

Before applying to the threshold-based measurement weighting strategy to the data, it is necessary to optimize its parameters* ad hoc*. To do it, small random datasets of 9 dimensions, which is the dimension of the real data used in the clustering analysis, are generated and a 2D grid search for the optimal parameters of the threshold-based weighting approach was performed as described previously. As shown in Figure 4, our result suggests that the optimal clustering result is achieved when setting *α* = 0.45 and *β* = 0.09, which will be used in the following analysis. Please note that, under this setting, around 45% of measurements are assigned with minimal weight in the analysis, most of which are likely to be located on very lowly expressed genes, whose methylation status cannot be reliably estimated. This is consistent with our knowledge that only around half of all the genes are expressed in a specific cell type [57]. Although penalizing these RNA methylation sites may inevitably repress some patterns, our experiments suggest the overall effect is to enhance the aggregation patterns of epitranscriptome modules and thus contribute to the clustering analysis.

A major difficulty for assessing the quality of the identified epitranscriptome module is the lack of ground truth. Although there exists bioinformatics database MetDB [58] supporting the query about epitranscriptome regulation of RNA methylation sites by enzymes, this evidence has not been properly integrated and a specific regulation may be supported by only a single study, lacking consistency between different experiments. Additionally, the known enzyme genes, including RNA methyltransferases METTL3, METTL14, WTAP and demethylase FTO, and ALKBH5, although have the potential may not actually play a leading regulatory role or induce an epitranscriptome module and it is very likely that there exist additionally still unknown regulators of the m^6^A epitranscriptome, such as the newly identified RNA m^6^A methyltransferase METTL16 [39, 59]. For the aforementioned reasons, it is difficult to provide a ground truth to assess the identified epitranscriptome modules; we thus consider an alternative approach by using gene ontology (GO) as a guidance; i.e., for two epitranscriptome modules that consist of the same number of genes, the one that has more GO terms more significantly enriched is more biologically meaningful and thus more likely to represent a true epitranscriptome module than the other one [60]. It is important to note that the two modules in comparison need to be of the same size, because a larger group is a lot more likely to have more GO terms enriched in it compared with a smaller group. Additionally, because the epitranscriptome modules identified from different approaches are likely to be of different size, in practice it is still difficult to compare the results from different methods under the aforementioned scheme. To solve this problem, we proposed an alternative indirect approach, in which the epitranscriptome modules identified from clustering analysis are directly compared with random modules of the size using GO analysis. If the modules identified from one approach are a lot more likely to be more biological meaningful than the random modules, while that identified from a different approach is less likely to be more biological meaningful than the random modules, then the former approach, which generated more biological meaningful results, may be considered superior to the latter one.

Specifically, 3,000 out of the total of 10000 RNA methylation sites after preprocessing are randomly selected, to which hierarchical clustering analysis was applied with or without measurement weighting strategy. The gene ontology enrichment analysis was conducted based on human gene ontology annotation database downloaded from R package org.Hs.eg.db [61] on Bioconductor. All of the three GO categories (BP, CC, and MF) were used in the enrichment analysis with the 3,000 random selected methylation sites set as the background. When calculating the biological significance of a specific epitranscriptome module, the GO terms with more than 1,000 counts in the background are considered too general and thus discarded from the analysis. The* p *values were calculated from one sided hypergeometric test for each GO term using customized R script, and the top 20 GO terms with the most significant* p* values were treated with negative logarithm and added together as the measurement of the biological significance of a specific module. As previously described, the clustering results (epitranscriptome modules identified) of different approaches are then compared indirectly via random gene set of the same size. Please note that we used in this analysis only a fraction (3000 sites) rather than all the 10000 RNA methylation site, which is essentially the bootstrap sampling strategy for achieving a more robust results. The previous analysis was repeated 100 times to rule out the possible impact of randomness. Because the optimal number of clusters is not available, we tested 3 different settings, i.e., the number of clusters *k* = 2, 5, and 10.

**Figure 5:**
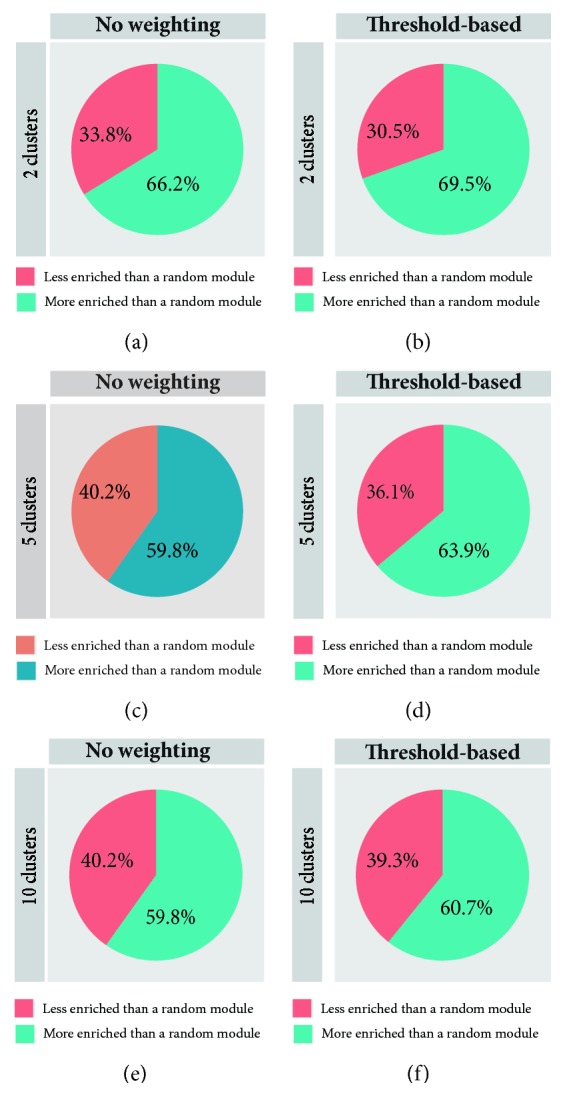
**Comparing epitranscriptome module detection based on biological significance**. The epitranscriptome modules identified from clustering analysis are always more likely to be biologically meaningful than the random modules, and this is true for clustering analysis using the measurement weighting strategy (66.2%, 59.8%, and 59.8% when *k* = 2, 5, and 10, respectively). The results obtained with measurement weighting scheme consistently outperform those obtained without measurement weighting (69.5% vs 66.2 when *k* = 2, 63.9% vs 59.8% when *k* = 5, and 60.7% vs 59.8% when *k* = 10), suggesting the proposed threshold-based measurement weighting strategy is helpful to improve clustering result and find more biological meaningful epitranscriptome modules. Clustering analysis with or without measurement weighting strategy was applied to 3000 random selected RNA methylation sites, and the epitranscriptome modules identified are compared with random group of genes of the same size in terms of biological significance using gene ontology enrichment analysis. Using bootstrap sampling approach, the analysis was repeated for 100 times and the results are summarized in this figure.

As is shown in Figure 5, the epitranscriptome modules identified from clustering analysis are always more likely to be biologically meaningful than the random modules and this is true for clustering analysis using the measurement weighting strategy (66.2%, 59.8%, and 59.8% when *k* = 2, 5, and 10, respectively) or not using the measurement weighting strategy (69.5%, 63.9%, and 60.7% when *k* = 2, 5, and 10, respectively), suggesting that the epitranscriptome module not only contains a number of RNA methylation sites whose methylation states are coregulated but also carries some biological significance that can be captured using gene ontology analysis. It is the first time to be proved true on real RNA methylation data with rigorous statistical analysis that the regulatory functions are enriched in epitranscriptome modules. The results obtained with measurement weighting scheme consistently outperform those obtained without measurement weighting (69.5% vs 66.2 when *k* = 2, 63.9% vs 59.8% when *k* = 5, and 60.7% vs 59.8% when *k* = 10), suggesting the proposed threshold-based measurement weighting strategy enhanced the clustering result and helped to find more biological meaningful epitranscriptome modules.

### 3.5. The Biological Functions of Epitranscriptome Modules

**Figure 6:**
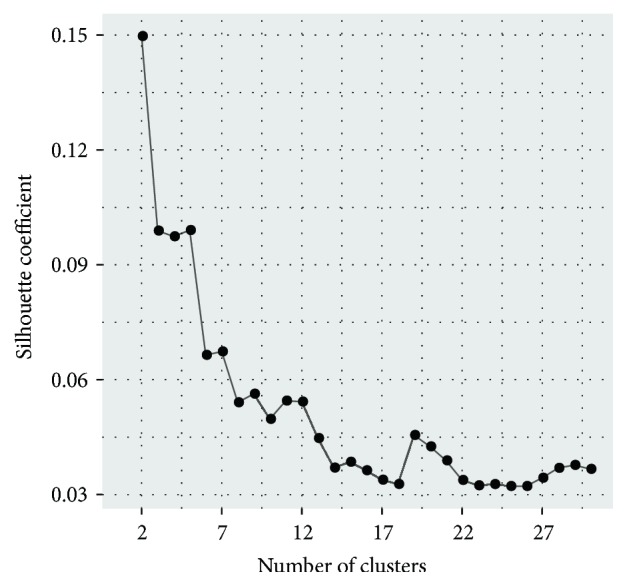
**Silhouette coefficient on the epitranscriptome data**. The Silhouette coefficient was used to assess the quality of clustering result when a different number of clusters are used (*k*). However, the largest value obtained is only 0.15, suggesting there is no clear evidence to support a specific model.

We, next, seek to explore the biological meanings of true epitranscriptome modules using the proposed measurement weighting strategy. Before clustering analysis is applied, we firstly try to use Silhouette approach [62] on all the preprocessed RNA methylation data to determine an optimal number of clusters. As shown in Figure 6, the largest Silhouette coefficient value obtained is only 0.15, suggesting there is no clear evidence to support a specific model (number of clusters). This is reasonable because that the epitranscriptome regulation is complex with multiple regulators and a single RNA methylation site can be regulated by multiple regulators simultaneously. Additionally, the epitranscriptome data is highly noisy due to the impact of transcriptome regulation and bias in sequencing. Even with the proposed approach, we may still miss true epitranscriptome modules or capture false positive patterns. Because an optimal number of clusters could not be determined, we set arbitrarily *k* = 5. The number was chosen to be not too small or too large for downstream functional analysis of the epitranscriptome modules identified.

**Figure 7:**
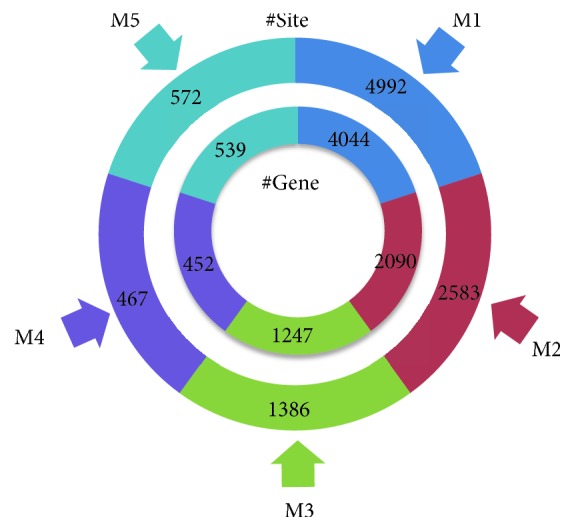
**Epitranscriptome modules identified from hierarchical clustering analysis**. Hierarchical clustering analysis of the RNA methylome identified 5 epitranscriptome modules with 4492, 2538, 1386, 467, and 572 sites, respectively, which are located on 4044, 2090, 1247, 452, and 539 genes.

We then applied hierarchical clustering (*k* = 5) with threshold-based measurement weighting strategy (*α* = 0.45 and *β* = 0.09) to the entire preprocessed data to search for epitranscriptome modules. As shown in Figure 7, clustering analysis identified 5 epitranscriptome modules with 4492, 2538, 1386, 467, and 572 sites, respectively, which are located on 4044, 2090, 1247, 452, and 539 genes. It is possible that multiple RNA methylation sites located on the same gene belong to the same or different epitranscriptome modules.

**Figure 8:**
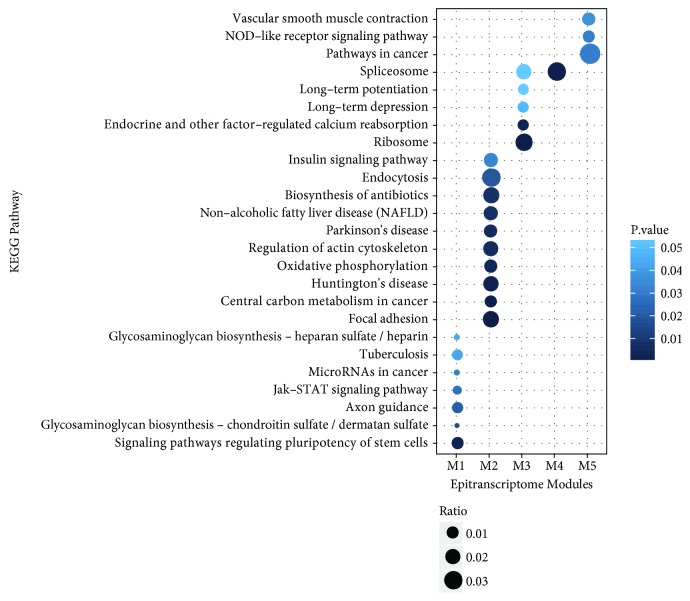
**KEGG pathways enriched in epitranscriptome modules**. Distinct pathways are enriched in different epitranscriptome modules. Interestingly and consistent with our understanding, insulin signaling pathway and nonalcoholic fatty liver disease are both enriched in epitranscriptome module M2 [48, 49] and axon guidance is enriched in module M1 [50]. Figure shows the top 10 most statistically enriched KEGG pathways in the identified epitranscriptome modules from DAVID [51]. Less terms are shown if there are less pathways enriched with significance level 0.05 using default setting of DAVID.

The five identified epitranscriptome modules (M1-M5) are then functionally annotated using DAVID website [51] to explore their biological relevance (the complete results are available in Supplement Materials Table S4.). Distinct KEGG pathways are enriched in the modules. Notably, Huntington's disease, Parkinson's disease, Alzheimer's disease, and synaptic vesicle cycle are all enriched in the identified epitranscriptome module M2, which is consistent with our understanding of the role of RNA methylation in neurological diseases [5, 63]. The circadian rhythm pathway, which has been shown to be regulated via the epitranscriptome [2], is enriched in epitranscriptome module M2. Many cancer related pathways are also overrepresented in different epitranscriptome modules, including, transcriptional misregulation in cancer, signaling pathways regulating pluripotency of stem cells, basal cell carcinoma and microRNAs in cancer enriched in M1, pathways in cancer, and small cell lung cancer enriched in M5. Besides, pathways related to obesity, such as insulin signaling pathway and nonalcoholic fatty liver disease, are also enriched in epitranscriptome module M2, suggesting a possible relationship with FTO, which is the first obesity-related gene identified from GWAs analysis [48] and the first known RNA m^6^A demethylase [49].  Figure 8 shows the most enriched KEGG pathways of each epitranscriptome module.

**Figure 9:**
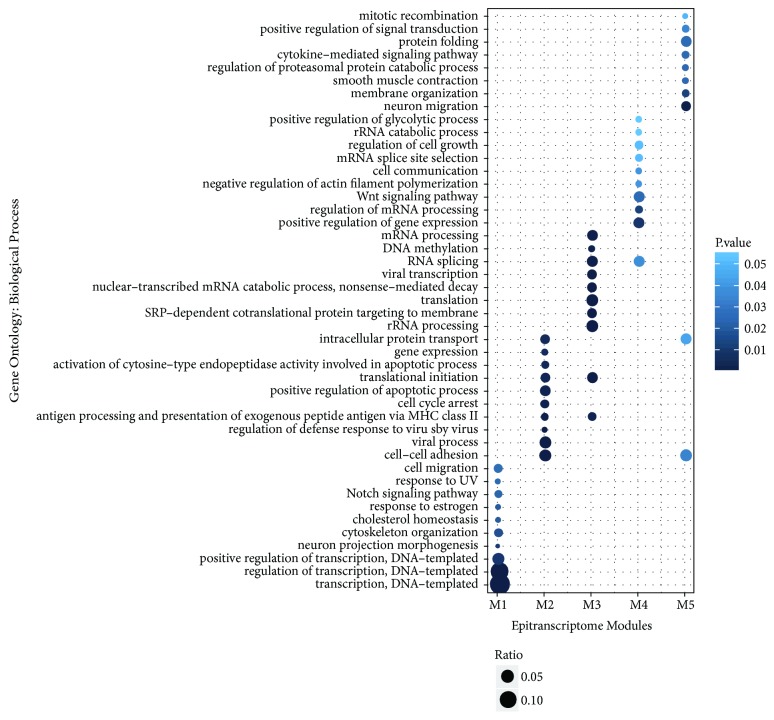
**Biological processes enriched in epitranscriptome modules**. Distinct biological processes are enriched in different epitranscriptome modules. Figure shows the top 10 most statistically enriched biological processes in the identified epitranscriptome modules from DAVID [51].

Besides the pathway-based enriched analysis, DAVID also reveals the association between gene ontology (GO) terms and the identified epitranscriptome modules (the complete results are available in Supplement Materials Table S4.).  Figure 9 shows the top 10 mostly enriched GO functions related to biological process. Interestingly, epitranscriptome module M1 is enriched with functions related to transcription (positive regulation of transcription); and M2 is enriched with positive regulation of apoptotic process, cell cycle arrest, regulation of defense response to virus, and viral process; M3 is enriched with mRNA/rRNA processing, RNA splicing, DNA methylation, and translation.

To test whether the identified epitranscriptome module is potentially induced by the activity of RNA methylation enzymes, we firstly identified the WTAP-dependent methylation sites in 3 different cell lines (A549, Hela, and HEK293T) using exomePeak R/Bioconductor package by performing differential RNA methylation analysis on MeRIP-seq data obtained from WTAP knockdown and wild type conditions [31, 33]. We then compared the identified WTAP target sites and the 5 identified epitranscriptome modules. Interestingly, we found that epitranscriptome module 1 is significantly enriched in WTAP preferential target sites under all 3 conditions (A549 cell line: Odds Ratio= 3.1910, p value = 5.87E-45; HeLa cell line: Odds Ratio= 3.7395, p value = 3.94E-28; and HEK293T cell line: Odds Ratio= 2.3401, p value = 8.22E-23), suggesting it is very likely to be mediated by WTAP, a very important component of m^6^A RNA methyltransferase protein complex [33].

## 4. Conclusion

Due to the impact of context-specific gene expression and limitation of sequencing depth, the epitranscriptome data is highly noise and it is usually difficult to accurately quantify the methylation level of very lowly expressed genes using conventional approaches developed for ChIP-seq or RNA-seq. In order to more accurately capture the epitranscriptome modules, which reflects the regulation imposed via epitranscriptome layer, we propose to use measurement weighting strategy to penalize the measurements that are less accurate due to weak signal in sequencing data. In this study, two different types of weighted schemes (logarithm-based & threshold-based) are developed. A 2D grid search was performed to further optimize the parameters of threshold-based approach. When the proposed measurement weighting strategy is applied under a hierarchical clustering approach, we show in real data that compared with conventional approach without a measurement weighting scheme, the proposed approach can indeed help to improve the classification performance and identify more biologically meaningful epitranscriptome modules. When applied to the real dataset using the optimal parameters determined from a training process, 5 epitranscriptome modules are identified from real data with distinct biological functions linked to recent studies in the field, suggesting the potential usage of the proposed method.

The proposed method is the first approach developed for dealing with RNA m^6^A epitranscriptome sites with low reads coverage in a clustering analysis. Although demonstrated under a hierarchical clustering analysis framework with Euclidean distance, the proposed measurement weighing strategy is conceptually easy and can be conveniently extended to another computational analysis related to distance measurement concerning the epitranscriptome and RNA methylation, such as, K-means, K-nearest neighbor methods, and Pearson correlation, related to RNA m^5^C methylome. For example, we show in the Supplementary Materials that the proposed threshold-based weighting scheme is equally applicable when using squared Euclidean distance or City Block to measure the similarity of RNA methylation profiles. The approach clearly pointed out that many measurements from high-throughput sequencing data may not be accurate and need to be handled carefully to keep as much information as possible and at the same time avoid possible contamination in signal.

It is worth mentioning that using 100 repeated experiments in a bootstrap sampling analysis, we show that the epitranscriptome modules are more likely to be biologically meaningful than a random group of genes of the same size in terms of gene ontology analysis. As far as we know, this is the first time to show with robust statistical analysis (rather than in a single isolated example) that the biological functions are enriched in epitranscriptome modules. Previously, epitranscriptome modules are considered the induced pattern of epitranscriptome regulators and are expected to emerge when a large number of RNA methylation sites are regulated by a small number of regulators [46], which explains the generation mechanism of epitranscriptome modules. Our results suggest that, besides the generation mechanism, the epitranscriptome modules also directly regulate corresponding biological functions, which justifies the regulatory aims of epitranscriptome modules. Our work established the functional basis of epitranscriptome modules, which fulfilled a key prerequisite for further functional characterization and deciphered the epitranscriptome and its regulation.

The study still has a number of limitations that may be improved. Firstly, the proposed threshold-based approach relies on two parameters that need to be optimized in data analysis. In practice, the most suitable values of the two parameters are likely to vary on different datasets, which may not be easy to determine in lack of appropriate training dataset. It would be nice to develop an easy-to-use parameter optimizing procedure for the proposed threshold-based approach or propose a nonparametric method. Secondly, due to the data availability and the lack of clear evidence for the optimal number of clusters, we explored the biological functions of epitranscriptome modules using only 9 samples and set the number of clusters *k* = 5; additionally, the clustering structure used assumes that the clusters identified are mutually exclusive; i.e., a methylation site can only belong to a single cluster. In practice, it is important to include more samples, using different number of clusters and different clustering structures such as biclustering to capture other potentially interesting epitranscriptome patterns. Thirdly, this study takes advantage of only the numeric patterns embedded in m^6^A-seq data [24, 25] but not data from other techniques such as CLIP-based approach [64] that may capture the direct target substrate of RNA methylation-related enzymes. An integrative analysis of multiple data types that address both the generation mechanism and the regulatory aims of the epitranscriptome modules is highly desired to paint a global picture of the epitranscriptome. It would be very interesting to see how a specific epitranscriptome enzyme, e.g., FTO, regulates a specific biological function via modulating the methylation status of thousands of substrate genes.

## Figures and Tables

**Table 1:** Datasets in the study.

Dataset ID	Tissue/Cell	Treatment	# Sample (IP & input)	Source
1	HepG2		4 & 3	[25]
2	HepG2	UV	1 & 1	[25]
3	HepG2	HS	1 & 1	[25]
4	HepG2	HGF	1 & 1	[25]
5	HepG2	IFN	1 & 1	[25]
**6**	Human Brain		1 & 1	[25]
**7**	HEK293T		3 & 3	[56]
**8**	U2OS		3 & 3	[2]
**9**	U2OS	DAA	3 & 3	[2]

**Table 2:** Percentage of correct clustering.

**Clustering Approach**	**# Trial**	**# Correct Result**	**% Correct Result**
Random Guess	916	30	3.22%
No weighting		241	26.31%
Logarithm-based weighting		294	32.06%
Threshold-based weighting		344	37.60%

## Abbreviation

m^6^A: N6-methyladenosine
MeRIP-Seq: Methylated RNA immunoprecipitation sequencing
IP: Immunoprecipitation
GO: Gene ontology
BP: Biological process.

## Supplementary Materials

Supplementary 1 In terms of the cases when M-value, squared Euclidean, or City Block is used to quantify the RNA methylation status or the similarity of RNA methylation profiles, the results are shown in the Supplementary Materials (Tables S1-S3 and Figures S1, S2).

Supplementary 2 The complete gene set enrichment analysis result of the 5 epitranscriptome modules identified from real dataset is available in Supplementary Materials Table S4.
