# Supplementary material for: Enhancing Epitranscriptome Module Detection from m6A-Seq Data Using Threshold-Based Measurement Weighting Strategy
Source: Biomed Res Int. 2018 Jun 14;2018:2075173. doi: 10.1155/2018/2075173 (PMC6022261; doi:10.1155/2018/2075173)
Supplement: Supplementary 1 — In terms of the cases when M-value, squared Euclidean, or City Block is used to quantify the RNA methylation status or the similarity of RNA methylation profiles, the results are shown in the Supplementary Materials (Tables S1-S3 and Figures S1, S2). [file 2075173.f1.docx]

Supplementary Materials

Enhancing epitranscriptome module detection from m^6^A-seq data using threshold-based measurement weighting strategy

**Table S1 Clustering performance when M-value is used to quantify RNA methylation level**

| **Clustering Approach** | **# Trial** | **# Correct Result** | **% Correct Result** |
| --- | --- | --- | --- |
| Random Guess | 916 | 30 | 3.22% |
| No weighting |  | 321 | 35.04% |
| Logarithm-based weighting |  | 341 | 37.21% |
| Threshold-based weighting |  | 393 | 42.90% |

Note: parameters $\alpha$ and $\beta$in the threshold-based weighting strategy is 0.45 and 0.03, respectively. The proposed threshold based approach can also improve clustering performance when M-value is used to quantify the RNA methylation level. Euclidean distance is used.

**Table S2 Clustering performance when using squared Euclidean distance**

| **Clustering Approach** | **# Trial** | **# Correct Result** | **% Correct Result** |
| --- | --- | --- | --- |
| Random Guess | 916 | 30 | 3.22% |
| squared Euclidean |  | 238 | 25.98% |
| Logarithm-based weighting |  | 295 | 32.20% |
| Threshold-based weighting |  | 367 | 40.07% |

Note: parameters $\alpha$ and $\beta$in the threshold-based weighting strategy is 0.45 and 0.03, respectively. The proposed threshold based approach can also improve clustering performance when Euclidean distance is used as distance measurement. Beta-value is used to quantify RNA methylation level.

**Table S3 Clustering performance when using City Block distance**

| **Clustering Approach** | **# Trial** | **# Correct Result** | **% Correct Result** |
| --- | --- | --- | --- |
| Random Guess | 916 | 30 | 3.22% |
| squared Euclidean |  | 258 | 28.16% |
| Logarithm-based weighting |  | 301 | 32.86% |
| Threshold-based weighting |  | 400 | 43.66% |

Note: parameters $\alpha$ and $\beta$in the threshold-based weighting strategy is 0.45 and 0.03, respectively. The proposed threshold based approach can also improve clustering performance when City Block is used as distance measurement. Beta-value is used to quantify RNA methylation level.

**Adding weights to Squared Euclidean and City Block**

The formula for the squared Euclidean distance is: , and the corresponding weighted squared Euclidean distance is: . The formula for city block distance is: , and the corresponding weighted city block distance is:

**
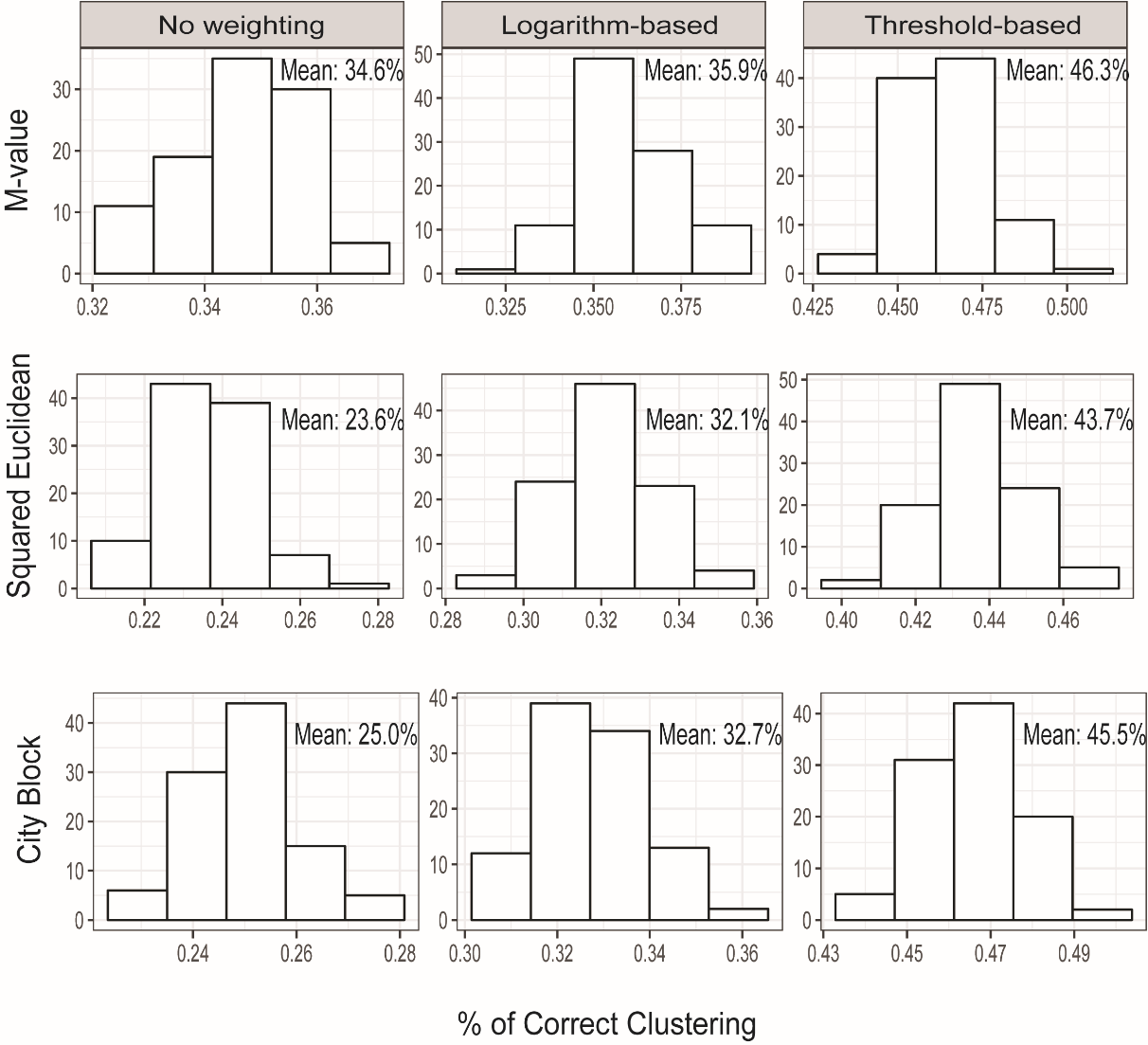
**

**Figure S1. Performance based on M-value, Squared Euclidean and City Black.** Similar to the case when Beta-value and Euclidean distance are used to quantify the RNA methylation level and the similarity of methylation profiles, the proposed weighting scheme can also improve significantly when M-value is used to quantify RNA methylation level, or Squared Euclidean or City Block is used to measure the similarity of methylation profiles. Parameters $\alpha$ and $\beta$in the threshold-based weighting strategy is 0.45 and 0.03, respectively.

**Figure S2. Impact of dimension size.**  Similar to the case when Beta-value and Euclidean distance are used to quantify the RNA methylation level and the similarity of methylation profiles, the proposed weighting scheme can also improve significantly when M-value is used to quantify RNA methylation level (**Figure S2a**), or Squared Euclidean (**Figure S2b**) or City Block (**Figure S2c**) is used to measure the similarity of methylation profiles. Parameters $\alpha$ and $\beta$in the threshold-based weighting strategy is 0.45 and 0.03, respectively.
